# Effect of a participatory multisectoral maternal and newborn intervention on maternal health service utilization and newborn care practices: a quasi-experimental study in three rural Ugandan districts

**DOI:** 10.1080/16549716.2017.1363506

**Published:** 2017-09-05

**Authors:** Elizabeth Ekirapa-Kiracho, Rornald Muhumuza Kananura, Moses Tetui, Gertrude Namazzi, Aloysius Mutebi, Asha George, Ligia Paina, Peter Waiswa, Ahmed Bumba, Godfrey Mulekwa, Dinah Nakiganda-Busiku, Moses Lyagoba, Harriet Naiga, Mary Putan, Agatha Kulwenza, Judith Ajeani, Ayub Kakaire-Kirunda, Fred Makumbi, Lynn Atuyambe, Olico Okui, Suzanne Namusoke Kiwanuka

**Affiliations:** ^a^ Department of Health Policy Planning and Management, Makerere University School of Public Health, Kampala, Uganda; ^b^ Unit of Epidemiology and Global Health, Department of Public Health and Clinical Medicine, Umeå University, Umeå, Sweden; ^c^ Department of International Health, Johns Hopkins Bloomberg School of Public Health, Baltimore, MD, USA; ^d^ School of Public Health, University of the Western Cape, Bellville, South Africa; ^e^ Makerere University Centre of Excellence for Maternal and Newborn Health Research, Kampala, Uganda; ^f^ Global Health Division, Karolinska Institutet, Stockholm, Sweden; ^g^ Kibuku District Health Office, Kibuku, Uganda; ^h^ Pallisa District Health Office, Pallisa, Uganda; ^i^ Kamuli District Health Office, Kamuli, Uganda; ^j^ Department of Obstetrics and Gynaecology, Makerere University Medical School, Kampala, Uganda; ^k^ Department of Epidemiology and Biostatistics, Makerere University School of Public Health, Kampala, Uganda; ^l^ Department of Community Health and Behavioural Sciences, Makerere University School of Public Health, Kampala, Uganda

**Keywords:** Maternal, newborn, participatory action research, community health workers, implementation science

## Abstract

**Background**: The MANIFEST study in eastern Uganda employed a participatory multisectoral approach to reduce barriers to access to maternal and newborn care services.

**Objectives**: This study analyses the effect of the intervention on the utilization of maternal and newborn services and care practices.

**Methods**: The quasi-experimental pre- and post-comparison design had two main components: community mobilization and empowerment, and health provider capacity building. The primary outcomes were utilization of antenatal care (ANC), delivery and postnatal care, and newborn care practices. Baseline (*n* = 2237) and endline (*n* = 1946) data were collected from women of reproductive age. The  data was analysed using difference in differences (DiD) analysis and  logistic regression.

**Results**: The DiD results revealed an 8% difference in early ANC attendance (*p* < 0.01) and facility delivery (*p* < 0.01). Facility delivery increased from 66% to 73% in the intervention area, but remained unchanged in the comparison area (64% vs 63%, *p* < 0.01). The DiD results also demonstrated a 20% difference in clean cord care (*p* < 0.001) and an 8% difference in delayed bathing (*p* < 0.001). The intervention elements that predicted facility delivery were attending ANC four times [adjusted odds ratio (aOR) 1.42, 95% confidence interval (CI) 1.17–1.74] and saving for maternal health (aOR 2.11, 95% CI 1.39–3.21). Facility delivery and village health team (VHT) home visits were key predictors for clean cord care and skin-to-skin care.

**Conclusions**: The multisectoral approach had positive effects on early ANC attendance, facility deliveries and newborn care practices. Community resources such as VHTs and savings are crucial to maternal and newborn outcomes and should be supported. VHT-led health education should incorporate practical measures that enable families to save and access transport services to enhance adequate preparation for birth.

## Background

Globally every year more than 303,000 women die because of pregnancy- and childbirth-related complications [1]. Of these deaths, 99% occur in developing countries and 66% in sub-Saharan Africa [1]. In Uganda, about 438 women die out of 100,000 live births each year because of pregnancy-related complications [2]. Most of these deaths could be averted using safe delivery care services [3]. Annually, about 40 million women worldwide give birth at home, putting their lives and the lives of their newborns at risk [4]. In Uganda, about 43% of women give birth at home [2].

The factors that hinder women from accessing these life-saving services have been commonly described in relation to the Three Delays Model [5]: delay in seeking care, delay in reaching facilities, and intra-institutional delay in providing timely and appropriate care. Delay in seeking care and delay in reaching care are caused by inadequate birth preparedness and delay in recognizing danger signs [6–11]; long distances to health facilities, compounded by poor transport and inability to afford transport costs [6,8–14]; and a preference for alternative traditional providers for prenatal or delivery care services [8,11]. Intra-institutional delay has been attributed to factors such as inadequate human resources for health, who are poorly motivated and may not have the appropriate skills set; poor attitudes towards pregnant women; lack of an enabling environment and inadequate infrastructure (equipment, theatres, electricity, ambulances) required to provide emergency obstetric care services; and lack of adherence to quality of care standards [6,8–12,15,16].

Low-cost interventions that can ensure safe delivery for women and their newborns are well documented [3,17,18] and are especially useful when provided during the 48 h surrounding labour and delivery within a continuum of care [17,18]. Strategies such as home visits by community health workers (CHWs), to increase awareness about the importance of maternal and newborn danger signs and the importance of seeking care at health facilities [16,19–21], have been used to address the first delay. The second delay has been mitigated by two main strategies, which aim to bring the services closer to those in need by providing home-based care, such as the use of CHWs and community midwives [14,20,21], and to improve access to transportation to allow the pregnant women to reach facilities more quickly. The latter includes strategies that provide easier access to cash, through the use of vouchers and conditional cash transfers, and community-based initiatives that improve the transportation itself [22–25]. Quality improvement initiatives such as maternal and newborn audits, monitoring labour, referral protocols and transport, are some of the strategies that have been used to reduce inter-institutional delays in providing care [6,16,22,26].

Community-based strategies that involve the training of CHWs and traditional birth attendants have resulted in increased awareness of maternal and newborn health and newborn danger signs, increased utilization of antenatal care (ANC) and facility delivery [1,17,20,27,28], and increased newborn care practices such as clean cord care, immediate breastfeeding, thermal care and delayed bathing [19,21]. Community mobilization and the use of community support groups have also resulted in increased awareness and knowledge about maternal and newborn health and increased facility deliveries [6]. Facility-based strategies that include provision of emergency obstetric care, training of health workers, provision of equipment, drugs and supplies, and refurbishment of facilities, have also had positive effects on facility delivery [3,16]. The effectiveness of these different intervention strategies depends on several factors that include the effectiveness of the intervention package, the implementation efficiency, and the availability of an enabling social and political environment [6,16]. Interventions often fail because they do not harness stakeholder resources across sectors.

To reduce the above constraints to comprehensive improvements in access to maternal health services, the Makerere University School of Public Health (MakSPH) implemented the Maternal and Neonatal Implementation for Equitable Systems project (MANIFEST) [21]. MANIFEST was a 3 year project (2013–2015) that used a participatory action research approach to tackle both demand- and supply-side constraints. More details about the intervention are provided in the study protocol paper in this special issue [29]. This paper aimed to determine the effect of this participatory multisectoral intervention on the utilization of maternal and newborn services and care practices in the intervention and comparison areas, in addition to determining the predictors of maternal service utilization and newborn care practices.

## Methods

### Study design and study area

This study employed a quasi-experimental pre- and post-comparison study design. It was implemented in the districts of Kamuli, Kibuku and Pallisa in eastern Uganda, with a total population of 1,075,242 in 2014 [30]. This population mostly practises subsistence farming, crop farming, petty trading and small-scale animal rearing. The whole of Kibuku district was an intervention area, because it has only one administrative zone, referred to as a health subdistrict. Kamuli and Pallisa have three administrative zones, and so one health subdistrict in each of these two districts was selected as an intervention area and one as a comparison area. The district team selected the intervention and comparison areas. The selection was purposive and determined based on maternal and newborn service indicators for the district. The health service infrastructure comprised a total of 104 health facilities, 33 in Pallisa, 17 in Kibuku and 54 in Kamuli.

### The MANIFEST intervention

The project had two main components: a community mobilization and empowerment component to stimulate demand for services, and a health provider and management capacity-building component to strengthen the delivery of quality maternal and newborn health services. The community mobilization and empowerment component comprised several strategies, including: (1) home visits by CHWs, also referred to as village health teams (VHTs); (2) health education through radio spots, talk shows and quarterly community dialogues; (3) promotion of saving through savings groups and other methods; and (4) promotion of partnerships with local transporters to ease geographical access to care. The capacity-building component included: (1) emergency obstetric and newborn care refresher training; (2) mentorship and support supervision of primary health workers; (3) a certificate course in health services management for health managers and a postgraduate diploma in project planning and management for district health officers; and (4) recognition of best performing facilities and managers. This supply-side package of interventions aimed to improve the skills of health workers in the provision of maternal and newborn care services, in addition to improving skills in leadership for maternal and newborn health care, to provide an enabling environment for service delivery. A detailed description of the intervention is provided in the design paper that is part of this supplement [29].

This intervention was provided in line with Susman’s participatory action research approach [31]. This approach comprised five main stages: (1) diagnosing, during which problems are identified; (2) action planning, during which alternative courses of action are considered and the best options selected; (3) taking action, during which selected courses of action are implemented; (4) evaluation, during which the actions taken and consequences are evaluated; and (5) specifying learning, during which key lessons are identified. Tetui et al. [32] provide a detailed description of the participatory approach used in this paper.

### Study variables

The primary outcomes for this paper were early ANC attendance (defined as ANC attendance in the first trimester); attending ANC at least four times; delivery in a health facility; postnatal care (PNC) attendance within 6 weeks; and newborn care practices, such as clean cord care (putting nothing on the umbilical cord), delayed bathing (bathing the newborn 24 h after birth) and skin-to-skin care. The independent variables included VHT home visits (visits by a VHT at home while pregnant or after delivery); community dialogue meeting attendance; receipt of health education about maternal and newborn health on the radio; saving for maternal health (saving money to meet maternal health-related needs); wealth (measured using a wealth asset index); and sociodemographic characteristics such as age, gender, marital status, educational level and occupation. The wealth quintiles were generated using principal components analysis based on the information collected on assets and household structure.

### Sample size determination and sampling procedure

The sample size was determined using a two-sided *Z *test of the difference between proportions (Equation (1)) with 80% statistical power, a 5% significance level, 1.5 design effect and a non-response rate of 10%. The major quantifiable outcome of the study used in the calculation of the sample size was the proportion of women who delivered in a health facility with a skilled provider. We therefore assumed that after 3 years (2013–2015) of implementation, skilled deliveries would increase from 38% to 58%, from 62% to 72% and from 68% to 78% in the intervention areas of Kibuku, Pallisa and Kamuli districts, respectively [21]. The assumptions resulted in a sample size of 2293 women.

(1) $$n=\frac{{\left(Z_{\alpha /2}+Z_{\beta }\;\right)}^{2}\left((\pi_{1}\left(1-\pi_{1}\right)+\pi_{2}\left(1-\pi_{2}\right)\right)}{{\left(\pi_{1}-\pi_{2}\;\right)}^{2}}$$

A two-stage sampling technique was applied per district for each of the study areas. We estimated that we required 119 villages to realize our sample size. Therefore, 52 out of 514 villages were selected for Kamuli, 46 out of 346 for Pallisa and 21 out of 244 for Kibuku using probability proportionate to size sampling techniques. Thereafter, all households were listed to identify eligible study participants. During listing, 3456 and 3199 women were identified as having delivered in the 12 months preceding the baseline and endline, respectively. The inclusion criteria comprised all women of reproductive age, who were residents and had delivered in the past 12 months, irrespective of birth outcomes (only pregnancies which lasted at least 28 weeks were considered). Women aged less than 18 years who met the inclusion criteria and provided informed consent were included as emancipated minors.

Women who were severely ill at the time of the survey and those who had not lived in the community for at least 1 year were excluded from the study. Of the women listed in the 119 villages, a total of 2237 (1101 in the comparison area and 1136 in the intervention area) were interviewed during the baseline survey and 1946 during the endline (920 in the comparison and 1026 in the intervention).

### Data collection

A detailed description of the data collection methods has been presented in the design paper [29]. The data were collected using interviewer-administered structured questionnaires in 2013 and 2015. The questionnaires were translated into local languages used in the respective districts to obtain data from the study participants in a language easily understood by them. Before data collection, the tools were pre-tested and adjusted according to the suggestions made by the pre-testing team. The data collection team comprised 24 research assistants, two editors and two field supervisors. They were trained and divided into two teams. All members of the data collection team were fluent in the local language and had completed secondary level education. The information collected included information about sociodemographic characteristics, places of birth, number of ANC and PNC attendances, pregnancy gestational age at the first ANC visit, birth preparedness, area of residence, newborn care practices, home visits by CHWs and participation in community dialogue meetings.

### Data management and analysis

A data collection manual outlined the procedures to be followed during data collection, storage and entry. To ensure that the data were collected accurately, the field supervisors reinterviewed randomly selected respondents, while the data editors checked for errors in the data collection forms. Any errors identified were verified and corrected immediately by the field staff. In addition, an independent quality control team visited the field every week to ensure that the data were being collected according to the set protocol. The data were entered into Epi info 7. To check the consistency of data entry, 10% of the questionnaires were double entered. The entered data were transferred into STATA 13.0 for analysis, and backed up.

Descriptive statistics of the independent and dependent variables are presented using frequencies. Difference in differences (DiD) analyses (Equation (2)) were used to understand the contribution of the intervention package towards health facility utilization and maternal and newborn care practices.

(2) $$y_{is}=\alpha +\beta_{1}treatment+\beta_{2}time+\beta_{3}(treatment\#time)+\lambda_{i}\sum_{i=1}^{n}x_{it}+\mu_{it}$$

The treatment and time variables were dummy variables: 1, treatment group; 0, non-treatment group; and 0, before intervention; 1, after intervention, respectively. $y_{is}$ represents the study outcomes, which included health facility delivery, ANC attendance, PNC attendance and newborn care practices. *β*_3_ is the DiD estimator that tells us whether the expected mean change in outcomes before the intervention and after the intervention were different in the intervention and control groups. $x_{it}$ represents covariates such as age, education and occupation, while $\lambda_{i}$ represents the covariates’ estimators. We ran the model separately for each of the study outcomes by considering all the covariates that we thought had an effect on the outcome variables. A significant coefficient of the interaction term implies that the outcomes differed by group over time.

Multivariate analysis was performed using logistic regression to understand the predictors of the study outcomes (newborn care practices, early ANC attendance, fourth ANC attendance and health facility delivery). We performed univariate analysis using *ulogit command* in STATA to seek the likelihood of covariate variables in affecting the study outcomes. Variables with *p* values ≤ 0.25 were considered for multivariate analysis. Multicollinearity was assessed using the *collin command* in STATA, where variables with large values of the variance inflation factor (> 2.0) were considered as strongly correlated factors and subsequently dropped from the final model. Hosmer–Lemeshow and Pregibon tests were used to test the goodness of fit of the model. A model was considered a good fit if the linktest (hatsq) under Pregibon’s test and *p* value under the Hosmer–Lemeshow test were non-significant. We introduced interaction terms between the VHT and area of study, and between saving for health and study area to assess how the VHT home visits and saving for health affected health utilization differently in the intervention and comparison areas. Similarly, we introduced the interaction between health facility delivery and study area to assess how health facility delivery affected newborn care practices differently in the intervention and comparison areas.

## Results

### Sociodemographic characteristics of the respondents

Table 1 summarizes the sociodemographic characteristics of women who participated in the baseline and endline surveys in the intervention and comparison areas. There were statistically significant differences in religion and education at the baseline. During the endline, the differences in religion persisted, while the differences in educational level were no longer statistically significant. However, the differences in occupation, which were not statistically significant at the baseline, were statistically significant at the endline.

**Table 1.** Sociodemographic characteristics of the women respondents.

	Baseline			Endline		
	Comparison	Intervention	*p*	Comparison	Intervention	*p*
Overall	1101 (100)	1136 (100)		920 (100)	1026 (100)	
Age group (years)						
14–19	168 (15.3)	163 (14.4)		138 (15.0)	149 (14.5)	
20–24	300 (27.3)	327 (28.8)	0.614	305 (33.2)	346 (33.7)	0.567
25–29	271 (24.6)	271 (23.9)		205 (22.3)	219 (21.4)	
30–34	202 (18.4)	191 (16.8)		153 (16.6)	155 (15.1)	
≥ 35	160 (14.5)	184 (16.2)		119 (12.9)	157 (15.3)	
Age (years)	26.5 ± 6.6	26.7 ± 7.1	0.266	26.12 ± 6.6	26.27 ± 6.5	0.769
Educational level						
None	715 (65.0)	819 (72.1)		574 (62.4)	638 (62.2)	
Primary	290 (26.4)	234 (20.6)	0.001***	269 (29.2)	293 (28.6)	0.773
Post-primary	95 (8.6)	83 (7.3)		77 (8.4)	95 (9.3)	
Parity						
≤ 3	275 (25.0)	264 (23.2)	0.325	421 (45.8)	487 (47.5)	0.452
≥ 4	825 (75.0)	873 (76.8)		499 (54.2)	539 (52.5)	
Occupation						
Salaried worker	28 (2.6)	29 (2.6)	0.408	17 (1.9)	27 (2.6)	0.001***
Business	51 (4.6)	40 (3.5)		63 (6.9)	35 (3.4)	
Peasant	1021 (92.8)	1068 (93.9)		840 (91.3)	963 (94.0)	
Religion						
Catholic	283 (25.7)	265 (23.3)		404 (43.9)	438 (42.7)	
Protestant	493 (44.8)	495 (43.5)		208 (22.6)	224 (21.8)	
Muslim	192 (17.5)	150 (13.2)	0.001***	170 (18.5)	161 (15.7)	0.001***
Pentecostal/Born Again	120 (10.9)	208 (18.3)		110 (12.0)	189 (18.4)	
Other	12 (1.1)	19 (1.7)		28 (3.0)	14 (1.4)	

Data are shown as *n* (%) or mean ± SD.

*** *p* < 0.0001.

### Effect of the intervention on maternal and newborn health facility utilization

The DiD results revealed an 8% difference in early ANC attendance (*p* < 0.01) with an increase of 8% in the intervention area and no change in the comparison area (29%) (*p* < 0.01). Attending at least four ANC visits increased by 12% and 7% in the intervention and comparison areas, respectively (*p* < 0.1) (Table 2).

**Table 2.** Effect of intervention on maternal health utilization and newborn care practices.

Indicators	Baseline (*n* = 2236)			Endline (*n* = 1946)			DiD
	C (%)	I (%)	Diff. (I – C)	C (%)	I (%)	Diff. (I – C)	
Health facility utilization indicators							
Early ANC attendance	29	25	−4*	29	33	4	8**
Attended ANC at least four times	53	51	−2	60	63	3	5
Delivered at the health facility	64	66	2	63	73	10***	8**
Woman received PNC services	52	58	6**	61	68	7**	1
Newborn received PNC services	53	62	9***	62	70	8***	−1
Newborn care practices							
Delayed bathing	1	1	0	11	19	8***	8***
Put nothing on the cord	35	27	−8***	21	33	12***	20***
Skin-to-skin	58	65	7**	85	85	0	−7**

C, comparison area; I, intervention area; Diff., difference; DiD, difference in differences; ANC, antenatal care; PNC, postnatal care.

*p < 0.05, **p < 0.01, ***p < 0.001.

There was an 8% difference in facility delivery at the endline (*p* < 0.01). Health facility delivery increased from 66% to 73% in the intervention, but remained unchanged in the comparison area (64% vs 63%, *p* < 0.01). At the baseline and endline, fast progress of labour was the most common reason given for not delivering in a health facility in both the intervention and comparison areas (Table 3). The second and third most common reasons were both related to geographical accessibility to services.

**Table 3.** Reasons for not delivering at health facilities.

Reason	Baseline			Endline			DiD
	C (%)	I (%)	Diff. (I – C)	C (%)	I (%)	Diff. (I – C)	
No transport means	14	9	−5*	14	9	5*	0
Facility too far	14	10	−4*	11	6	−5*	−1
Labour progressed too quickly	43	41	−2	40	41	1	3
Too expensive	2	3	1	2	1	1	0
Not necessary	3	3	0	4	4	0	0
Others^a^	2	2	0	2	2	0	0

C, comparison area; I, intervention area; Diff., difference; DiD, difference in differences.

^a ^Did not know where to go, services are poor, no one to care for children.

*p < 0.05.

There was a significant increase in PNC attendance by mothers and newborns in both the intervention and comparison areas. However, the 1% difference between the intervention and comparison areas was not significant.

### Effect of the intervention on newborn care practices

According to the DiD results, there was a 20% difference in clean cord care (*p* < 0.001). At baseline, significantly fewer women in the intervention area put nothing on the newborn’s umbilical cord compared to those in the comparison area (27% vs 35%, *p* < 0.001), while at the endline more women in the intervention area put nothing on the newborn’s cord (33% intervention vs 21% comparison, *p* < 0.001) (Table 2). There was an 8% difference in delayed bathing (*p* < 0.001). Regarding newborn bathing, at baseline, delayed bathing was very low in both the intervention and non-intervention areas (both 1%, *p* > 0.05). However, at the endline, delayed bathing was significantly higher in the intervention area than in the comparison area (19% vs 11%, *p* < 0.001).

### Predictors for maternal health utilization

Table 4 provides detailed information on the predictors of early ANC attendance, fourth ANC attendance and skilled delivery. Women aged 14–34 years were more likely to attend ANC in their first trimester compared to those aged 35 years and above. Similarly, women who were residents of the intervention area and were visited by VHTs were 49% more likely to attend their first ANC in their first trimester compared to those who were residents of the comparison area and were not visited by the VHTs. Women who belonged to the poorest (first) wealth quintile were 65% less likely to attend ANC in their first trimester compared to those who belonged to the least poor (fifth) wealth quintile.

**Table 4.** Predictors of health facility utilization using logistic regression.

	Model 1	Model 2	Model 3
	Early ANC attendance	Attended ANC at least four times	Health facility delivery
Age group (years)			
14–19	1.69 (1.05–2.72)*	1.31 (0.84–2.05)	0.57 (0.35–1.01)
20–24	2.05 (1.39–3.03)***	1.61 (1.12–2.32)*	0.66 (0.44–1.07)
25–29	1.57 (1.10–2.24)*	1.64 (1.18–2.28)**	0.66 (0.47–1.03)
30–34	1.50 (1.03–2.19)*	1.37 (0.99–1.93)	0.79 (0.55–1.13)
≥ 35	1.00	1.00	1.00
Parity			
1–3	1.00	1.00	1.00
≥ 4	1.12 (0.84–1.49)	1.05 (0.79–1.40)	0.73 (0.54–0.97)*
Educational level			
None	1.00	1.00	1.00
Primary	0.96 (0.77–1.21)	1.15 (0.92–1.44)	1.21 (0.96–1.51)
Post-primary	0.91 (0.61–1.37)	1.23 (0.82–1.85)	1.13 (0.75–1.69)
Occupation			
Paid work	1.00	1.00	1.00
Peasant	1.08 (0.69–1.70)	0.87 (0.55–1.37)	0.77 (0.50–1.19)
Married			
No	1.00	1.00	1.00
Yes	1.03 (0.73–1.45)	1.25 (0.88–1.78)	1.08 (0.76–1.55)
Religion			
Pentecostal and others	1.00	1.00	1.00
Catholic	0.87 (0.63–1.19)	1.36 (1.01–1.85)*	0.94 (0.68–1.29)
Muslim	1.03 (0.73–1.44)	0.97 (0.70–1.35)	0.71 (0.51–1.01)
Protestant	1.04 (0.78–1.37)	1.14 (0.86–1.49)	0.91 (0.68–1.20)
Got information from radio			
No	1.00	1.00	1.00
Yes	1.50 (1.01–2.24)*	0.78 (0.53–1.17)	0.83 (0.56–1.23)
Wealth index			
1 (poorest)	0.65 (0.47–0.88)**	0.62 (0.46–0.85)**	1.22 (0.89–1.68)
2	0.74 (0.54–1.02)	0.85 (0.63–1.15)	1.06 (0.77–1.45)
3	1.10 (0.82–1.47)	1.02 (0.75–1.39)	0.92 (0.68–1.25)
4	0.83 (0.61–1.13)	0.77 (0.57–1.04)	0.94 (0.70–1.28)
5	1.00	1.00	1.00
Attended community dialogue			
No	1.00	1.00	1.00
Yes	0.69 (0.42–1.14)	1.57 (0.96–2.56)	0.91 (0.57–1.47)
Received VHT visits while pregnant × Study area interaction^a^			
Did not receive VHT visit × Comparison	1.00	1.00	1.00
Received VHT visit × Intervention	1.49 (1.01–2.19)*	1.09 (0.74–1.60)	–
Received VHT visit × Comparison	1.27 (0.90–1.78)	1.04 (0.75–1.45)	–
Did not receive VHT visit × Intervention	1.14 (0.78–1.66)	0.84 (0.58–1.23)	–
Attended ANC early^b^			
No	–	1.00	–
Yes	–	3.12 (2.49–3.90)***	–
Attended ANC at least four times			
No	–	–	1.00
Yes	–	–	1.42 (1.17–1.74)***
Saved money for health × Study area interaction^c^			
Did not save × Comparison	–	–	1.00
Saved money × Intervention	–	–	2.11 (1.39–3.21)***
Saved money × Comparison	–	–	1.13 (0.85–1.50)
Did not save × Intervention	–	–	1.77 (1.13–2.77)*
Model diagnostic tests			
*Mean VIF*	1.65	1.65	1.67
*_hat*	0.29	0.01	0.04
*_hatsq*	0.41	0.08	0.53
*Chi2(p)*	1191.90 (0.34)	1424.13 (0.23)	1432.62 (0.20)

Data are shown as adjusted odds ratio (95% confidence interval).

ANC, antenatal care; VHT, village health team.

a^ ^Omitted under skilled delivery model (Model 3) because of multicollinearity.

b^ ^Omitted under skilled delivery model (Model 3) because its *p* value was greater than 25% in univariate analysis.

c^ ^Omitted early ANC attendance (Model 1) and attended ANC at least four times (Model 2) because of multicollinearity.

*p < 0.05, **p < 0.01, ***p < 0.001.

Women who started their first ANC in their first trimester were at least three times more likely to attend ANC four times or more compared to those who did not start their first ANC in their first trimester. Women who belonged to the poorest (first) wealth quintile were 62% less likely to attend ANC at least four times compared to those who belonged to the least poor (fifth) wealth quintile.

Women who attended ANC at least four times were 42% more likely to deliver at the health facility compared to those who did not attend ANC at least four times. The interaction between saving for health and study area indicated that women who saved for maternal health and were residents of the intervention area were at least two times more likely to deliver in the health facility compared to those who did not save and were residents of the comparison area. Women with parity of at least four were 27% less likely to deliver at a health facility compared to those with parity below four.

### Predictors of newborn care practices

The predictors of newborn care practices are presented in Table 5. Women who had attained post-primary education were two times more likely to apply nothing on the newborn’s cord compared to those who had no education. Women who were visited by the VHT after delivery were 29% more likely to put nothing on the cords of the newborns compared to those who were not visited by the VHT after delivery. Similarly, women who delivered at the health facility in both the intervention and comparison areas were more likely to apply nothing on the newborn’s cord compared to women who did not deliver at the health facility in the comparison area.

**Table 5.** Predictors of newborn care practices using logistic regression.

	Model 1	Model 2	Model 3
	Put nothing on the cord	Delayed bathing	Practised skin-to-skin
Age group (years)			
14–19	0.58 (0.36–0.96)*	1.29 (0.69–2.40)	1.07 (0.56–2.06)
20–24	0.63 (0.42–0.94)*	0.94 (0.55–1.61)	1.25 (0.73–2.14)
25–29	0.85 (0.60–1.20)	0.98 (0.61–1.56)	1.29 (0.79–2.10)
30–34	0.96 (0.66–1.37)	1.49 (0.94–2.38)	0.92 (0.57–1.50)
≥ 35	1.00	1.00	1.00
Educational level			
None	1.00	1.00	1.00
Primary	1.21 (0.96–1.54)	1.28 (0.95–1.71)	1.08 (0.78–1.48)
Post-primary	2.04 (1.39–3.00)***	2.09 (1.37–3.18)***	0.67 (0.41–1.08)
Parity			
1–3	1.00	1.00	1.00
≥ 4	0.95 (0.69–1.29)	0.81[0.54–1.20)	1.22 (0.80–1.86)
Married			
No	1.00	1.00	1.00
Yes	0.81 (0.55–1.18)	1.29 (0.79–2.10)	1.05 (0.65–1.71)
Occupation			
Paid work	1.00	1.00	1.00
Peasant	0.92 (0.60–1.41)	1.18 (0.70–1.91)	1.01 (0.59–1.73)
Religion			
Pentecostal and others	1.00	1.00	1.00
Catholic	0.82 (0.60–1.14)	0.96 (0.64–1.45)	0.97 (0.62–1.52)
Muslim	1.12 (0.80–1.59)	1.74 (1.15–2.62)**	1.13 (0.70–1.84)
Protestant	0.81 (0.61–1.08)	0.88 (0.61–1.27)	0.81 (0.54–1.20)
Wealth index			
1 (poorest)	0.90 (0.65–1.26)	0.82 (0.55–1.23)	1.20 (0.79–1.82)
2	1.02 (0.74–1.42)	0.62 (0.41–0.94)*	1.64 (1.06–2.55)*
3	0.99 (0.72–1.36)	0.84 (0.58–1.24)	1.11 (0.75–1.66)
4	0.961[0.70–1.33)	0.94 (0.64–1.37)	1.32 (0.87–1.99)
5	1.00	1.00	1.00
Visited by VHT after delivery			
No	1.00	1.00	1.00
Yes	1.29 (1.02–1.62)*	1.16 (0.87–1.55)	1.38 (1.01–1.90)*
Attended community dialogue			
No	1.00	1.00	1.00
Yes	1.10 (0.67–1.84)	0.94 (0.51–1.73)	1.03 (0.51–2.08)
Got information from radio			
No	1.00	1.00	1.00
Yes	1.14 (0.74–1.75)	1.17 (0.70–1.95)	1.20 (0.68–2.11)
Delivered at health facility × Study area interaction			
Did not deliver at health facility × Comparison	1.00	1.00	1.00
Delivered at health facility × Intervention	2.02 (1.31–3.12)**	2.21 (1.278–3.81)**	3.18 (1.85–5.46)***
Delivered at health facility × Comparison	1.43 (1.01–2.01)*	1.10 (0.707–1.72)	3.70 (2.50–5.47)***
Did not deliver at health facility × Intervention	1.36 (0.83–2.24)	1.34 (0.731–2.45)	0.63 (0.36–1.11)
Model diagnostic tests			
*Mean VIF*	1.87	1.87	1.87
*_hat*	0.01	0.01	0.35
*_hatsq*	0.76	0.39	0.28
*Chi2 (p)*	1425.51 (0.08)	1446.83 (0.06)	1344.61 (0.45)

Data are shown as adjusted odds ratio (95% confidence interval).

ANC, antenatal care; VHT, village health team.

*p < 0.05, **p < 0.01, ***p < 0.001.

Women who had attained post-primary education were two times more likely to practise delayed bathing compared to those who had no education at all. The odds of delayed bathing were at least two times higher among women in the intervention area who had delivered at the health facility compared to women in the comparison area who had not delivered at the health facility.

Regarding skin-to-skin care, women who delivered at the health facility in both the intervention and comparison areas were at least three times more likely to practise skin-to-skin care compared to those who did not deliver at the health facility in the comparison area. Also, women who were visited by VHTs after delivery were 38% more likely to practise skin-to-skin care compared to those who were not visited by VHTs after delivery.

## Discussion

These results have shown that a participatory multisectoral approach can lead to improvements in maternal and newborn service uptake and practices along the continuum of care during pregnancy, childbirth and the postnatal period. This approach led to increased early ANC attendance, increased fourth ANC attendance and increased facility deliveries. In addition, it led to significant changes in delayed bathing and clean cord care.

### Predictors of early ANC attendance

Early ANC attendance allows early detection of complications and appropriate management of these complications [33,34]. Being aged 14–34 years, being in the fourth or fifth wealth quintile and home visits by VHTS in the intervention area predicted early ANC attendance in this study. The association between an inability to attend ANC early and belonging to the poorest (first) wealth quintile is probably related to the fact that financial barriers are one of the factors that hinder early utilization of ANC services [35–37]. The VHTs, on the other hand, are likely to have convinced the women to attend ANC early by providing health education about the importance of early ANC attendance [38]. Ignorance about the importance of attending ANC early, and the gestational age at which women should attend their first ANC, is one reason that has been given for delayed attendance at ANC [35,37]. Moreover, work carried out in Uganda has shown that the main reason that many women give for attending ANC is to collect an antenatal card, which they can use during delivery, rather than to check on their own well-being and that of the baby [39]. Furthermore, Kisuule et al. showed that many women hear about the timing and importance of attending ANC early when they go for ANC at the health facility [35]. Women who have never been pregnant or who go for their first ANC late may therefore not be aware of this. The VHTs who are based in the communities can, therefore, play a vital role in increasing health education about the importance of attending ANC early, especially for such women [35]. These VHTs, however, need to have the appropriate skills if they are to have a positive influence. The results showed that VHT visits influenced early ANC attendance in the intervention area but not in the comparison area, suggesting that the VHT programme was more effective in the intervention area.

### Predictors of facility delivery

According to the multivariate analysis, the intervention elements that predicted facility deliveries included attending ANC four times and saving for maternal health. Saving for maternal health was encouraged as a means of preparing for birth to enable families to meet financial costs during delivery [30,40]. This is in keeping with several studies that have shown that lack of finances is one of the factors that can hinder facility delivery [15,41]. Facility delivery and VHT home visits were the main predictors for newborn care practices such as delayed bathing, clean cord care and skin-to-skin care. This was probably because health workers and VHTs educate newly delivered women and households about these practices. This increased awareness enhances the implementation of positive practices and aids in reducing delay in deciding to seek care [6,8,38]. Furthermore, VHTs are trusted by the community and so their messages are often more acceptable [6]. However, it is important to note that the influence of VHT home visits is likely to be affected by factors such as their selection, training, supervision, workload and incentives provided [42,43]. Countries that intend to use VHTs therefore need to ensure that these factors are taken into consideration.

### Factors that influenced intervention outcomes

We believe that several factors contributed to the positive outcomes seen in this intervention. These factors were related to the intervention package, its distribution and buy-in from the local stakeholders. According to Darmstard et al. [44], these three factors influence the effectiveness of a strategy.

Interventions for addressing maternal and newborn health issues are more likely to have positive outcomes if they comprise a package of interventions, rather than a single intervention [16,45].

Consequently, we decided to implement a package of intervention elements based on existing evidence of their effectiveness [16,17,19,20,27], feasibility of implementation and acceptability by the local stakeholders. Although evidence around the effectiveness of community-based savings groups and locally organized transport systems is more limited than evidence about strategies such as the use of CHWs, the literature shows that such arrangements have been included in intervention packages that have contributed to improved access to maternal healthcare services [6,45,46].

### Use of a participatory approach

The participatory approach selected for the implementation enhanced the use of locally acceptable methods of distribution for the different aspects of the intervention, since the district- and community-level stakeholders participated in selecting the approaches that were used. Furthermore, since the local stakeholders were responsible for the implementation of the project, they could take decisions to modify aspects that they felt needed modification. This participatory approach also promoted social approval and ownership of the project, which reduces resistance to the uptake of interventions [6].

### Cultural beliefs

Other authors have emphasized the scope of the intervention, implementation efficiency, availability of resources, leadership and local contextual issues as key factors that influence the successful implementation of interventions [6,28]. Local contextual cultural factors were noted to be vital in changing newborn care practices. We believe that newborn care practices such as delayed bathing and putting nothing on the cord are strongly influenced by embedded cultural beliefs that support these practices [8]. The reversal of such newborn care practices requires behavioural change, which usually takes longer than the 3 years for which the project was implemented. Implementers should, therefore, work with local cultural and opinion leaders, as well as VHTs who can play a key role in changing cultural beliefs that encourage negative practices such as immediate bathing and placing of harmful substances on the newborn’s cord.

### Strengths and limitations of the study

One of the strengths of this paper is that it presents the effects of an intervention that draws together multiple stakeholders from health, transport and finance sectors in addition to bringing together communities, local political and opinion leaders, and technocrats to address the demand- and supply-side constraints that hinder the utilization of maternal and newborn health services, using a participatory approach. One of the limitations of this paper is that the intervention was presented as a package. It was therefore not possible to separate the effects of the different components of the intervention. Some of the findings may have been affected by recall bias, although we believe that events around birth are key events and that the respondent is often able to remember them. Another factor that may have influenced our findings is the short implementation period (3 years); interventions that call for a change in behaviour often require longer time-frames. Lastly, the use of a quasi-experimental design may not have taken care of confounders that are often best handled through randomization.

## Conclusions

This multisectoral intervention contributed to early ANC attendance, increased fourth ANC attendance, facility delivery and improved newborn care practices. The intervention elements that predicted facility delivery included attending ANC four times and saving for maternal health. On the other hand, facility delivery and VHT home visits were key predictors for clean cord care and skin-to-skin care.

To achieve a positive influence on maternal and newborn outcomes, the provision of information about birth preparedness needs to be accompanied by practical measures that facilitate families to save and access transport services to enhance adequate preparation for birth. Therefore, multisectoral approaches that allow such arrangements should be encouraged by implementers and funders of health programmes. The participatory multisectoral implementation approaches are beneficial because they enhance local buy-in, provide a pool of multiskilled implementers and allow continued partnership with local stakeholders such as cultural and opinion leaders, as well as VHTs, who can play a key role in changing strongly held cultural beliefs that encourage negative practices, such as immediate bathing and placing harmful substances on the newborn’s cord.

## Paper context

Maternal and newborn mortality is still high in developing countries despite the existing interventions. This multisectoral intervention contributed towards early ANC attendance, increased facility delivery and improved newborn care practices. The intervention elements that predicted facility delivery included attending ANC four times and saving for maternal health. VHT-led health education should incorporate practical measures that facilitate families to save to enhance adequate preparation for birth.
